# Metastatic cutaneous squamous cell carcinoma accounts for nearly all squamous cell carcinomas of the parotid gland

**DOI:** 10.1007/s00428-024-03798-5

**Published:** 2024-04-17

**Authors:** Patrick J. Bradley, Göran Stenman, Lester D. R. Thompson, Alena Skálová, Roderick H. W. Simpson, Pieter J. Slootweg, Alessandro Franchi, Nina Zidar, Alfons Nadal, Henrik Hellquist, Michelle D. Williams, Ilmo Leivo, Abbas Agaimy, Alfio Ferlito

**Affiliations:** 1 Department of Otolaryngology Head and Neck Surgery, Nottingham University Hospitals, Queens Centre Campus, Nottingham, UK; 2 Department of Pathology, Sahlgrenska Center for Cancer Research, University of Gothenburg, Sahlgrenska University Hospital, Gothenburg, Sweden; 3 Head and Neck Pathology Consultations, Woodlands Hills, CA 91364 USA; 4 Sikl’s Department of Pathology, Faculty of Medicine in Pilsen, Charles University, Pilsen, Czech Republic; 5 Bioptic Laboratory, Ltd, Pilsen, Czech Republic; 6 Department of Anatomical Pathology, University of Calgary, Calgary, Alberta Canada; 7 Department of Pathology, Nijmegen Medical Centre, Radboud University, Nijmegen, The Netherlands; 8 Department of Pathology, Kilimanjaro Christian Medical University College, Moshi, Tanzania; 9 Department of Translational Research and New Technologies in Medicine and Surgery, University of Pisa, 56126 Pisa, Italy; 10 Institute of Pathology, Faculty of Medicine, University of Ljubljana, 1000 Ljubljana, Slovenia; 11 Department of Pathology, Hospital Clinic, Barcelona, Department of Basic Clinical Practice, School of Medicine, Universitat de Barcelona, Barcelona, Spain; 12 Faculty of Medicine and Biomedical Sciences, University of Algarve, Campus de Gambelas, Ala Norte, 8005-139 Faro, Portugal; 13 Algarve Biomedical Center Research Institute (ABC-RI), Faro, Portugal; 14 Department of Cellular Pathology, Northern Lincolnshire and Goole NHS Foundation Trust, Lincoln, UK; 15 Department of Pathology, The University of Texas M. D. Anderson Cancer Center, Houston, TX USA; 16 Institute of Biomedicine, Pathology, University of Turku, Turku, Finland; 17 Institute of Pathology, University Hospital Erlangen, Friedrich‐Alexander University Erlangen‐Nürnberg (FAU), Comprehensive Cancer Center (CCC) Erlangen-EMN, Erlangen, Germany; 18 Coordinator of the International Head and Neck Scientific Group, Padua, Italy

**Keywords:** Salivary gland carcinoma, Primary parotid squamous cell carcinoma, Metastatic cutaneous squamous cell carcinoma, Diagnostic misclassification, Cancer registration, UV mutational signature

## Abstract

Primary squamous cell carcinoma of the parotid gland (pSCCP) has long been recognized as a separate entity and is included in the WHO classifications of salivary gland tumors. However, it is widely accepted among head and neck pathologists that pSCCP is exceptionally rare. Yet, there are many publications describing series of pSCCP and data from SEER and other cancer register databases indicate erroneously an increasing incidence of pSCCP. Importantly, pSCCP and metastatic (secondary) squamous cell carcinoma to the parotid gland (mSCCP) have nearly identical histological features, and the diagnosis of pSCCP should only be made after the exclusion of mSCCP. Moreover, all of the histological diagnostic criteria proposed to be in favor of pSCCP (such as, for example, dysplasia of ductal epithelium) can be encountered in unequivocal mSCCP, thereby representing secondary growth along preexistent ducts. Squamous cell differentiation has also been reported in rare genetically defined primary parotid carcinomas, either as unequivocal histological squamous features (e.g., NUT carcinoma, mucoepidermoid carcinoma), by immunohistochemistry (e.g., in NUT carcinoma, adamantinoma-like Ewing sarcoma, basal-type salivary duct carcinoma, mucoepidermoid carcinoma), or a combination of both. Another major issue in this context is that the International Classification of Diseases (ICD) coding system does not distinguish between primary or metastatic disease, resulting in a large number of patients with mSCCP being misclassified as pSCCP. Immunohistochemistry and new molecular biomarkers have significantly improved the accuracy of the diagnosis of many salivary gland neoplasms, but until recently there were no biomarkers that can accurately distinguish between mSCCP and pSCCP. However, recent genomic profiling studies have unequivocally demonstrated that almost all SCCP analyzed to date have an ultraviolet light (UV)-induced mutational signature typical of mSCCP of skin origin. Thus, mutational signature analysis can be a very useful tool in determining the cutaneous origin of these tumors. Additional molecular studies may shed new light on this old diagnostic and clinical problem. This review presents a critical view of head and neck experts on this topic.

## Introduction

Salivary gland cancers (SGCs) are rare, representing < 5% of all malignancies of the head and neck region and are morphologically heterogeneous [1]. Since the early 1970s, significant research has improved our understanding of the nature of SGCs, applying diverse methods of analysis that encompass conventional light microscopy, electron microscopy, immunohistochemistry (IHC), and molecular genetic testing. It has been emphasized with each revision of the WHO classification that the histologic classification of SGCs is dynamically evolving with the introduction of new entities and refinement and reclassification of existing ones [2, 3]. This is evident from the first WHO classification of SGCs in 1972 in which 7 histological types of carcinoma were listed, while in the most recent WHO classification from 2022, 21 different malignant salivary gland tumor entities were included [2, 4], in addition to many subtypes. As a consequence, retrospective reviewing of SGCs by hospitals or national datasets has resulted in reclassification of as many as 30% of salivary gland carcinomas [5–7]. Reclassification results in changes not only from one cancer type to another of the same or different type and grade but also from a primary to a metastatic (secondary) tumor or from a malignant to a benign lesion [8]. This finding emphasizes that a thorough histological revision is necessary in the reporting of any retrospective study to ensure accurate diagnosis and allow for reliable evaluation of the frequency of the different tumor types [7].

Despite the dramatic achievements in the precise classification of the majority of primary SGC cases over time, the inclusion of primary SCC has persisted in a less critical way, yet clinicians refer to the diagnostic conundrum with the inability to distinguish histopathologically between primary and metastatic (secondary) SCC [9, 10]. A report of the SGCs data of the Surveillance, Epidemiology, and End Results (SEER) Program (USA) between 2011 and 2016 shows that the most frequent diagnoses in a series of 9722 patients were SCC (*n* = 2101) and mucoepidermoid carcinoma (MEC) (*n* = 2093). In the elderly population (65 years +), a diagnosis of SCC accounted for 32% (1695/5315) and MEC for 14% (738/5315) [11]. The consensus opinion among salivary gland histopathologists is that SCC of the parotid gland is exceptionally rare, and that making such a diagnosis “when the lesion is an epithelial neoplasm with exclusive squamous differentiation can only be done after the exclusion of metastatic SCC” [12–14]; the later being almost impossible to rule out reliably in many cases due to the advanced age of many of the patients and the lack of a complete clinical history.

## Primary squamous cell carcinoma of the parotid gland (pSCCP)

**Fig. 1:**
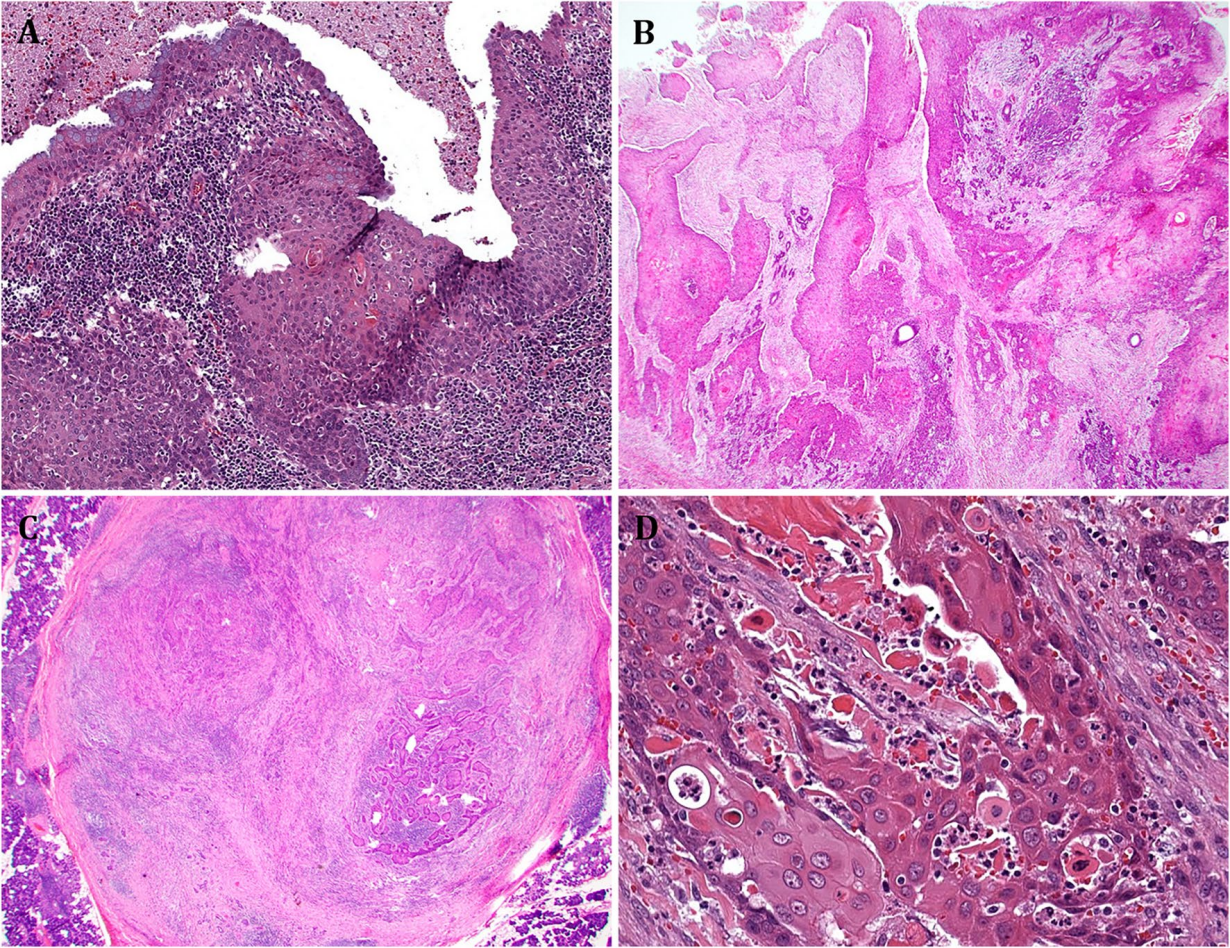
**A** An exceptionally rare example of pSCCP showing clear-cut transition from normal ductal epithelium to squamous metaplasia and carcinoma in situ associated with stromal invasion. **B** Contiguous invasion of the parotid gland by cutaneous SCC shows cystic (duct-like) area lined by carcinoma tissue that may be mistaken for dysplastic ductal epithelium. **C** Nodular circumscription of intraparotid lymph node metastasis from cutaneous SCC. Advanced lesions may not show clear-cut lymph node remnants. **D** Conventional SCC histology at high power

It has been proposed that primary SCC of the salivary glands originates via squamous metaplasia and dysplasia of the ductal epithelium with subsequent progression to SCC [10, 15–17] (Fig. 1 A). Metaplasia may be caused by chronic trauma due to sialadenitis, sialolithiasis, and/or prior radiotherapy [18, 19]. This is even more true in the submandibular gland, where sialolith-induced squamous metaplasia of major ducts is a frequent finding, but dysplasia is exceptional. This arc of development is exceptionally rare. Histological documentation of sialodochodysplasia must be present to prove pSCCP. The major issue with this notion is the fact that genuine dysplasia unassociated with overt invasive SCC is seldom, if ever, encountered in routine evaluation of the thousands of salivary gland specimens seen in large busy centers. This is also true in the submandibular gland, where sialoliths are much commoner than in the parotid. In line with this, the possible premalignant changes in salivary ductal squamous epithelium never represent a topic in the head and neck pathology literature. This is in contrast to many other organs, such as the urinary bladder where the squamous phenotype is not rarely seen in dysplastic urothelium or in situ carcinoma; the invasive component of which frequently displays SCC morphology.

## Why is the parotid gland a major target for metastatic (secondary) squamous cell carcinoma (mSCCP)?

The embryology of the parotid gland is unique among the major salivary glands in that numerous lymph nodes are incorporated within and adjacent to the glandular parenchyma [20]. These lymph nodes receive most of the lymphatic drainage from the facial skin and anterior scalp, as well as the parotid salivary parenchyma. Because of this unique anatomy, the parotid gland has been described as a “metastatic basin” for metastatic skin cancers and is most often affected by metastasis from cutaneous SCC (cSCC) (Fig. 1 B–D) and melanoma [21]. In addition, extension of primary or recurrent cSCC into the parotid is common. Metastasis from a cutaneous facial malignancy to the parotid gland lymph nodes is most frequent, but metastasis from mucosal SCC of head and neck sub-sites (oral cavity, oropharynx, sinonasal tract, nasopharynx, larynx, hypopharynx, and conjunctiva) has been reported only rarely [22]. Cancers of organs located below the clavicles, such as the breast, kidney, prostate, and lung, have also been shown to metastasize to the lymph nodes and salivary parenchymal tissue of the parotid gland [23].

cSCC is one of the commonest malignancies, with most cases presenting on the sun-exposed (ultraviolet [UV] radiation) skin of the head and neck region [24]. About 5% of cSCC metastasize [25, 26] to regional lymph nodes: 46/75 had parotid gland metastasis versus 42/75 with metastasis to lymph nodes only [26]. High-risk sites include ear, retroauricular, cheek, and lip [27]. In-transit metastases (lymphatic deposits) are most common in head and neck SCC [27], from the scalp, forehead, and temple. Basins of lymph node involvement for head and neck tumors cannot be reliably predicted, and thus issues about skip lesions (supraclavicular lymph nodes instead of pre-auricular for a forehead primary) cannot be reliably assessed.

Recent data from Germany and East Coast USA have shown an increasing incidence of metastatic cSCC to the parotid gland [28–30]. Most cases present in males (more than 80%) aged > 65 years [28–30]. The exact incidence of cSCC is unknown as cSCC is often not included in Tumor Registries [31, 32] since it is such a common malignancy (similar to cutaneous basal cell carcinoma). However, a recent estimate indicates that there were 2.2 million cases of cSCC globally in 2015 [33]. The annual increase in global incidence of cSCC is estimated to be 5–6% [32]. Approximately 55% of all head and neck cSCC (HNcSCC) occur on the facial skin with high-risk disease being more common. High-risk factors include primary site (ear, temple, hair-bearing lip), clinical size, and immune status. In the USA, around 2500 people die annually with regional nodal metastasis from cSCC [33]. The specific factors that predispose to lymph node metastasis from cSCC include primary tumor depth of invasion (thickness), tumor size, proliferation index, perineural invasion, poorly differentiated, sarcomatoid and undifferentiated tumor, and significant desmoplasia.

Although primary cSCC is common, metastasis develops in only about 5% [25, 26, 33], but this might be underestimated as many patients presenting with parotid SCC metastases years after cSCC might not have been included or codified as such. Indeed, this underestimation of cSCC metastases is likely responsible for the overestimation of pSCCP in other studies. The parotid gland is the most common site of metastasis and are concurrent in some 20% of the patients at presentation, while in other cases, the presentation of intra-parotid metastasis usually occurs 1–3 years after surgical removal of the primary [34]. An Australian study on the impact of location of nodal metastasis on survival showed that disease in the neck resulted in worse disease specific survival (DSS) and overall survival (OS) compared to isolated parotid disease, and that patients with multiple parotid gland lymph nodes affected had a similar clinical course as those with a single involved neck node. Patients with multiple cervical nodes deposit had the shortest survival [35].

## Miscoding of mSCCP versus pSCCP in cancer registries and population-based studies is a major source of statistical bias

The SEER data between 1973 and 2009 [36], which showed an increase in salivary gland cancer, included a wide range of morphological codes for SCC (8070/2 SCC in situ, 8070/3 conventional SCC, 8052/3 papillary SCC, 8078/3 to 8083/3 basaloid SCC, and 8084/3 clear cell SCC). The authors concluded that the apparent increase in incidence was due to parotid cancers diagnosed as SCC. They also questioned the accuracy of the results since they are limited by the accuracy of the information entered into the database. Two additional SEER publications (data from 1988 to 2009) [37, 38] came to a similar conclusion that the data wholly depends on the registration/codification process and that it is difficult to control the reliability of certain aspects of the data. It was also impossible to independently confirm the accuracy of the histological diagnosis without access to primary patient data.

Other authors, when analyzing primary carcinomas of the major salivary glands from the population-based cancer register (Landeskrebsregister LKR) in North Rhine-Westphalia in Germany over a 10-year period, excluded the data on SCC, defined as ICD-0–3 morphology codes 8050/3–8084/3, from analysis as the information required for the differentiation between primary and secondary SCC was not “sufficiently available” [39]. This group also investigated whether the large percentage of primary parotid SCC recorded could be due to misclassification of a metastatic tumor and found that most cases were indeed misclassified metastatic parotid cancers of cutaneous origin [40]. A major review of the incidence of major SGC in England, between 1990 and 2013 (11,432 new cases registered), found that the incidence rate had risen from 0.94 cases per 100,000 inhabitants during 1990–1994 to 1.25 cases in 2009–2013. Analyses of the histological diagnoses revealed that 1972 cases (17%) were SCC. This was interpreted as indication that the majority of cases did not originate in the salivary glands but were metastatic cancers from other sites [41]. In summary the ICD-10 coding system does not distinguish between a primary or a metastatic SCC when diagnosed in the parotid gland and can therefore not be used to track pSCCP versus mSCCP. Notably, most of the head and neck pathologists involved in the current review have not observed genuine pSCCP cases in their files that collectively includes several thousands of salivary gland carcinomas. In one of the authors registries (A. S.), that encompasses mostly consultation cases, 81 parotid gland tumors were coded as “squamous cell carcinoma” among 1506 parotid gland carcinomas, but only one case was coded as likely primary, indicating an overall frequency of putative pSCCP among parotid gland carcinomas of approximately 0.07%.

## Primary squamous cell carcinoma of the parotid gland is a diagnosis of exclusion that is exceptionally rare

Traditionally, pSCCP has been considered a diagnosis of exclusion. Indeed, it was Foote and Frazell who originally questioned its existence, raising the notion that pSCCP may represent diffuse squamous overgrowth occurring in a MEC [16]. If fine needle aspiration cytology (FNAC) of a parotid gland mass shows SCC, such cases warrant investigation as a carcinoma of unknown primary (CUP) [42, 43]. The diagnosis of pSCCP requires that other malignancies are excluded, including mucosal and cutaneous SCC as well as other primary salivary gland malignancies, such as, for example, MEC and salivary duct carcinoma (SDC) [44, 45]. As MEC is one of the most common primary malignancies, standard evaluation is based on essential histomorphologic features seen on hematoxylin and eosin (H&E)-stained slides along with mucicarmine or other histochemical techniques used for demonstration of mucin production. Additional immunohistochemistry studies including p40/p63, CK5/6, androgen receptor, GCDFP-15, and CK7 (among others as necessary) may guide the diagnosis. Confirmation of MEC by detecting genomic rearrangements (gene fusions) of *MAML2* helps exclude SCC [46–48]. SDC (other than the basal phenotype) is nearly always reactive with the androgen receptor and most have focal GCDFP-15 reactivity [49].

## Genomic profiling of parotid, oral, and head and neck cutaneous SCC

Distinguishing head and neck mucosal SCC from a metastatic CUP is challenging. When in level II lymph nodes, a p16 positive SCC is assumed to be of oropharyngeal origin, but it is well recognized that SCC from other anatomic sites of origin may also express p16. In cases without transcriptionally active HPV, it is more challenging to document the primary site. cSCC may now be proven by identification of a UV light-related DNA damage signature, characterized by mutations with C to T transitions at dipyrimidine sites [50]. Mutational signatures of UV light exposure are not typically found in mucosal SCC. In comparison to cSCC, mucosal SCC have far lower tumor mutational burden and a much smaller fraction of mutations with C to T substitutions. A retrospective study was recently undertaken on 71 patients who had treatment for SCC involving the parotid gland between 2000 and 2018. A UV mutational signature was present in all 23 patients with a history of cSCC and in two of eight without a history of cSCC, while absent in three of three patients with a history of mucosal SCC [51]. The study highlighted how a UV signature may document a cSCC metastasis, with consequences for prognosis and treatment. The authors concluded that a combination of clinical history and genomic sequence analysis can help confirm a primary cutaneous source of SCC involving the parotid gland. Still, UV negativity does not exclude metastatic/direct extension from a mucosal site. These observations were recently supported by a large study comparing the genomic landscape of oral and head and neck cutaneous SCC [52]. The study confirms that a high tumor mutational burden in combination with a UV signature is typical for cSCC and may guide future treatment decisions, including the use of immunotherapy.

## Critical state-of-the-art approach in exclusion of pSCCP

**Fig. 2:**
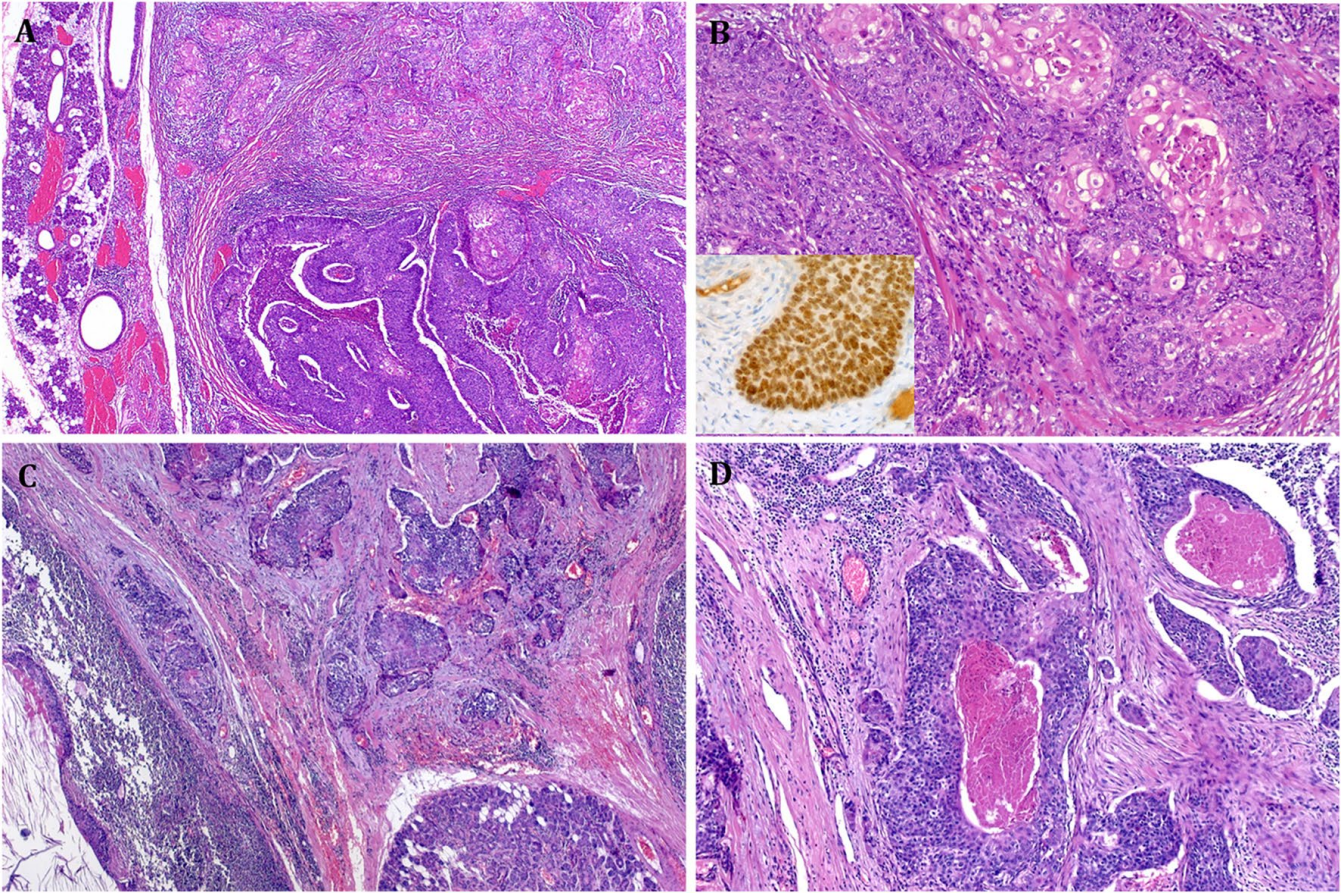
Examples of mimics of pSCCP. **A**, **B** Primary *BRD4::NUTM1* fusion-positive NUT carcinoma of the parotid closely mimicking SCC. The abrupt squamoid islands as a hint to the entity. **B** (inset) Strong nuclear reactivity for the monoclonal NUT antibody. **C**, **D** Primary SCC-like carcinoma of the parotid in a young adult showing variable poroid cytology and harboring an *YAP1::MAML2* fusion, consistent with skin-analog squamoid porocarcinoma (clinical examination and whole-body imaging excluded any other primary)

When incorporating the current knowledge, exclusion of pSCCP seems not that simple as reflected in the older literature. Besides metastatic cSCC, a variety of entities and mimics need to be reliably excluded. These include neoplasms with overt focal squamous differentiation including, in particular, myoepithelial carcinoma, NUT carcinoma and adamantinoma-like Ewing sarcoma all showing abrupt squamous foci/morules, NUT, or MEC carcinoma of the parotid with variable squamous cell carcinoma morphology and rare squamoid porocarcinomas (Fig. 2 A–D). All these entities are either defined or can be verified by molecular markers such as *PLAG1* fusions in myoepithelial carcinoma [53], *EWSR1::FLI1/ERG* fusions in adamantinoma-like Ewing sarcoma, *NUTM1* fusions in NUT carcinoma [54], *MAML2* fusions in MEC, *YAP1::MAML2* fusion in squamoid porocarcinoma, and others [55] (Table 1). Likewise, squamous cell features in basally differentiated salivary duct carcinoma (SDC) can be reliably ruled out by thorough sampling to detect invasive or intraductal conventional SDC or evidence of a preexisting pleomorphic adenoma. Based on these observations, it became clear that the mere presence of diffuse and strong expression of high molecular weight keratins and p63/p40 is not enough to call a malignant neoplasm SCC. Notably, it is mainly general surgical pathologists, who are often less familiar with these uncommon mimics, who tend to name any unclassified keratin-positive malignancy SCC, merely based on a squamous immunophenotype.

**Table 1:** General features and genetic markers of the major mimics of pSCCP

Tumor type	Similarity to pSCCP	Genetic markers
Metastatic cutaneous SCC	May colonize preexistent salivary ducts, diffuse expression of squamous cell markers	UV mutational signature, *TP53* mutations
Metastatic non-cutaneous SCC	May colonize preexistent salivary ducts, diffuse expression of squamous cell markers	Frequent *TP53* mutations, no UV signature
Basal-type SDC	Variable, usually no conventional SCC features, variable expression of squamous cell markers	Frequent *TP53* mutations, intraductal SDC? Preexistent PA?
Myoepithelial carcinoma	Mimic basaloid SCC with abrupt squamous foci/morules, variable expression of squamous cell markers	+ / − *PLAG1* fusions, rarely *HMGA2*
NUT carcinoma	Mimic basaloid or conventional SCC, abrupt squamous foci may be present in 40% of cases, diffuse expression of squamous cell markers	*NUTM1* rearrangements
Adamantinoma-like Ewing sarcoma	Mimic basaloid SCC with abrupt squamous foci/morules, diffuse expression of squamous cell markers	*EWSR1::FLI1* fusions, rarely *FUS* or other fusion variants
Rare carcinomas with squamous phenotype	May show skin adnexal-like features	Limited data
Sclerosing microcystic adenocarcinoma	May mimic poorly cohesive SCC	Limited data

*SCC*, squamous cell carcinoma; *pSCCP*, primary squamous cell carcinoma of parotid; *SDC*, salivary duct carcinoma

After exclusion of all these morphological and/or immunophenotypic mimics of pSCCP, one is then confronted with the great challenge of conventional SCC in the parotid. Particularly in the elderly population, no single or set of histological features would be reliable to call a conventional SCC a primary parotid neoplasm. Molecular profiling to illustrate a UV mutational signature in such cases represents a powerful tool to predict metastatic cSCC. Finally, the question remains, whether it is more appropriate to call such a case in the parotid of an elderly metastatic SCC of unknown primary (CUP) as it is widely accepted in lymph node disease and to treat it as such? In the appropriate clinical (older age, mostly male sex) and pathological (conventional, mostly high-grade diffusely invasive SCC pattern) context, the latter approach seems more appropriate than rendering a putative diagnosis of pSCCP.

## Conclusions

The consensus opinion among salivary pathologists is that pSCCP is vanishingly rare and, in the experience of many, even almost non-existent and that this diagnosis should only be made very restrictively after the exclusion of metastatic SCC, in particular, head and neck cSCC. Its frequency, especially epidemiological registry-based data (SEER, NCDB, and institutional series), is not accurate nor clinically supported. Therefore, it is strongly recommended that the ICD be updated to include a specific code for primary and metastatic squamous cell carcinoma, just as there are metastatic codes for lymph nodes (ICD-11, 2D60.0, malignant neoplasm metastasis in lymph nodes of head, face, or neck), rather than just ICD-11 2B67.1 for squamous cell carcinoma of parotid gland or 2B68.1 for squamous cell carcinoma of submandibular or sublingual gland. Most studies conclude that the large percentage of primary SCC of the parotid gland is due to misclassified metastatic (secondary) parotid gland cancers, most frequently of cutaneous origin. This is further supported by recent genomic profiling studies showing that almost all parotid SCCs analyzed to date show a UV mutational signature typical of cSCC. Such studies may also identify new molecular targets for therapy and guide treatment decisions in metastatic settings. Refined current histological and molecular diagnostic criteria also help to exclude other carcinomas and sarcomas that can easily be mistaken for pSCCP. Establishing a new TNM staging approach to mSCCP of unknown or unverified origin in the elderly might be valuable in clinical oncological terms.
